# Montreal Veterinary Medical Association

**Published:** 1897-04

**Authors:** B. A. Sugden

**Affiliations:** Secretary-Treasurer


### MONTREAL VETERINARY MEDICAL ASSOCIATION.

THE regular meeting was held on the evening of January 28, 1897, and was presided over by the President, Dr. Baker. Dr. Martin was also present.

Mr. Thayer furnished an interesting report of a case of colic, which was treated as such, and the animal resumed work on the following morning. Two days later the animal was found to be suffering from another attack, the symptoms of which were those of an ordinary colic, with the exception of the temperature, which was subnormal, and the fact that the patient stood backed up in the corner of the box. As time went on the animal showed all the signs of enteritis, with the exception of the temperature, which was still not elevated. An ounce each of chloral hydrate and aromatic spirits of ammonia was given in a quart of oil. Subsequently two doses of sulphate of morphia of two and a half grains each were given hypodermatically, and hot woollen blankets applied to the abdomen. On passing the syringe into the rectum for the purpose of administering an enema an obstruction was encountered, which, on backraking the animal, proved to be sand. The animal died about midnight. A post-mortem examination showed that the intestines contained a large quantity of undigested food and sand, and that the floating colon was entirely obstructed, the amount of sand removed from it being some fifty-seven pounds. Discussion elicited the fact that sand was used for litter in the stable and that the animal was accustomed to eat dirt whenever he had an opportunity.

Mr. Stevenson then read a paper on "Parturition." This he confined almost entirely to a consideration of the normal act, which he divided into four stages, viz.: (1) Preliminary, (2) dilatation of the os, (3) expulsion of the fœtus, (4) expulsion of the membranes. These varying stages were then graphically described by the essayist, and after the subject had been discussed the chairman called upon Mr. Matthews for his paper on "Peritonitis."

In considering this subject the essayist divided it into two main classes —acute and chronic—and the acute into primary and secondary. Primary peritonitis is rare and may follow cold, but more modern views scarcely recognize cold as more than a predisposing cause, which permits the action of various micro-organisms. Secondary inflammation is due to extension from other organs, in which case the determining cause is the penetration into the peritoneal cavity of irritating germs. After considering the various pathological appearances Mr. Matthews said that there were probably essential differences between the various kinds of peritonitis, and that bacteriology was beginning to give valuable information upon this point; the organism most commonly present is the streptococcus pyogenes, and in a majority of the cases arising from perforation the bacillus coli communis is also present. The symptoms were next described, both in the horse and ox; but, as they have nothing typical, the clinical picture does not possess anything expressive, the numerous variations so often observed being due to its frequently secondary nature. The greatest diversity of opinion exists among authors, some advocating the use of purgatives, others condemning them. Both local and internal remedies may be employed. In small animals the application of cold may, in the primary stages, aid in combating the inflammation. In the larger animals turpentine stupes are more suitable. In suppurative peritonitis the exudate may be removed and the serous membrane washed out with an antiseptic solution. Personally, Mr. Matthews preferred the use of opium to purgatives, enemas for relieving the bowel, and in addition the administration of diuretics. In traumatic cases free drainage should be given and antiseptics carefully used. Mr. Matthews closed his paper with a few observations on chronic cases and their treatment.

In the course of a few remarks Dr. Martin pointed out that in the human subject the administration of opium would mask certain symptoms which might indicate the necessity of an operation, which on this account might be delayed with unfortunate results to the patient. Before closing the meeting Dr. Baker drew attention to the fact that peritonitis was seldom diagnosticated in the lower animals unless there was some history of previous injury.

A regular meeting was held on the evening of February 11, 1897, the President, Dr. Baker, occupying the chair. Dr. Duncan McEachran and Dr. Dawes were also present. Owing to his approaching departure from Montreal, the Librarian tendered his resignation to the Society. This was accepted with much regret, and a vote of thanks to Dr. Thurston for the excellent order in which the library had been kept during his term of office was carried unanimously. Mr. Spanton was then elected to fill the vacant position.

The case report of the evening was furnished by Mr. Spanton, and this proved to be one of exceptional interest. A cocker spaniel, about a year old, got its tail jammed in a doorway. On Mr. Spanton making an examination he found the last two joints frightfully mutilated, and thinking it best to remove them, he did so. As it was not convenient for the dog to be at liberty he was tied up, to which he strongly objected, and a couple of hours later he was found to be hanging back and trying to slip the collar. When seen the next morning his neck was found to be much swollen and very tender; the collar was therefore removed and he was secured to the chain by means of a bandage put around the forelegs and tied over the shoulder. Hot fomentations were applied, but by evening the swelling had much increased and respiration had become a matter of great difficulty. Thinking that the animal needed immediate relief, Mr. Spanton made a tracheotomy-tube out of the handle of a metal tea-pot, and by making an incision half-way down the neck inserted the tube in the usual manner. This was kept in for three days; when removed the wound was stitched with silk, the result of the operation being that the animal made a good recovery.

Dr. Baker complimented Mr. Spanton upon his ingenuity and the success of his operation, and after the case had been discussed called upon Mr. Moore for his paper. This was entitled "Breeding and Care of Dairy Cattle."

Mr. Moore at once defined good breeding as improving the stock from generation to generation in order to produce milk in larger quantities and of better quality at the least possible cost. This must be done by selecting the best males and females and mating them. A reliable opinion as to the value of a cow can only be formed from an exact record of her actual performances; but as this can rarely be obtained, certain external signs, such as the shape of the body and udder, have often to be depended upon. In choosing a sire he should invariably be pure bred, good in his own points, and if, in addition, he has good pedigree, so much the better. In-breeding must not be carried too far, or it will be certain to do harm, the progeny will be predisposed to disease, the males often impotent, and the females barren. While speaking of the best breeds for dairy purposes, the essayist put in a strong claim for the Ayrshire, and then went on to describe the care of stock, which included feeding, watering, stabling, and general management. After discussing the properties of various foods Mr. Moore drew attention to the following standard, which appeared in the *American Dairyman:* Total organic matter, 24.51; digestible protein, 2.5; digestible carbohydrates, 13.77; fat, .74; total digestible matter, 16.2 pounds. The function of water as regards animals is a most important and vital one. In the first place, there must be a sufficiency of water or you cannot maintain animal life, and Mr. Moore laid great emphasis on the fact that this water must be free from pollution if we wish our animals to be healthy and vigorous and their products wholesome. It must be recollected that past immunity does not necessarily imply a pure supply, and, further, that the action of water is most insidious, and there can be little doubt that many cases of diarrhœa and indigestion may be attributed to its effects, these often being the forerunners of more serious disorders. Before closing his paper Mr. Moore gave some valuable hints upon stabling and general management.

In the discussion which followed Dr. Dawes, in replying to a question on the subject, said that five pounds was about the most profitable quantity of ground meals that could be used, and that this must be mixed with some coarser food. Brewers' waste might be used if fed under proper conditions. In compounding a ration the price of various foods and the object for which you are feeding (for milk or for butter) must be considered. In reply to a question as to how long a cow should be milked, Mr. Moore thought that up to a month or six weeks before calving was quite sufficient. Dr. McEachran said that prairie-cows weaned their calves when about five months old, provided they were able to do so, and that in this province some dairymen allow their cows to be dry for four to five months, and the tendency to become dry early might possibly become hereditary. When closing the meeting, Dr. Baker thanked Mr. Moore for having brought such an interesting and important subject before the Society. Dairying was undoubtedly at the head of agricultural interests, and offered a wide field to the members of the profession. Many questions had been asked that evening which could not be answered off-hand; but in selecting a milch-cow, in addition to other signs, one should note the size and length of the milk-vein, and whether its course was straight or tortuous. In his opinion it was advisable to feed cattle three times a day, and whenever the weather permitted they should be let out. Water should be given in the barn, and if it was possible for them to have it constantly before them, so much the better.

The meeting then adjourned.

B. A. SUGDEN,
Secretary-Treasurer.

